# Hyperactivation of nuclear receptor coactivators induces PERK-dependent cell death

**DOI:** 10.18632/oncotarget.24451

**Published:** 2018-02-08

**Authors:** Muhammad Mosaraf Hossain, David Barua, Vahid Arabkari, Nahidul Islam, Ananya Gupta, Sanjeev Gupta

**Affiliations:** ^1^ Discipline of Pathology, School of Medicine, Lambe Institute for Translational Research, National University of Ireland Galway, Galway, Ireland; ^2^ Regenerative Medicine Institute (REMEDI) at CÚRAM Centre for Research in Medical Devices, School of Medicine, College of Medicine, Nursing and Health Sciences, National University of Ireland Galway, Galway, Ireland; ^3^ Discipline of Physiology, School of Medicine, National University of Ireland Galway, Galway, Ireland

**Keywords:** steroid receptor coactivators, unfolded protein response, breast cancer, PERK, NCOA3

## Abstract

Nuclear receptor coactivators (NCOAs) function as coactivators for nuclear receptors as well as several other transcription factors and potentiate their transcriptional activity. NCOAs play an important role in biology of hormone-dependent and -independent cancers. MCB-613 is a recently described, small molecule stimulator of NCOAs and anti-neoplastic compound that leads to the death of tumour cells due to increased cellular stress. In the present study we investigated the molecular mechanism of MCB-613-induced cell death. We report that absence of NCOA3 leads to compromised activation of PERK signalling pathway during unfolded protein response (UPR). We found that chemical and genetic inhibition of NCOA3 attenuated the expression of PERK at mRNA and protein level. We show that loss of NCOA3 renders cells hypersensitive to UPR induced cell death. Our results show that MCB-613 induced cell death is attenuated in NCOA3 knockout HeLa cells and MCB-613 leads to enhanced PERK signalling in wild-type HeLa cells. The knockdown of PERK provides resistance to MCB-613 mediated cell death while knockdown of XBP1 and ATF6 have no such effect. Our results suggest that hyperstimulation of NCOA3 by MCB-613 induces cell death by evoking constitutive PERK signalling. Taken together our results point to NCOA3 as an important determinant in regulating cell fate during ER stress, with too little and too much NCOA3 both producing deleterious effects.

## INTRODUCTION

Nuclear receptor coactivators (NCOAs) are members of p160 family of coactivators that collaborate with nuclear receptors (NR) and other transcription factors to regulate gene expression [1]. NCOAs were the first of the gene families to be identified and classified as coactivators for NRs. The NCOA family consists of three members: NCOA1 (also known as SRC-1), NCOA2 (also known as SRC-2, GRIP1, and TIF2) and NCOA3 (also known as SRC-3, ACTR, AIB1, p/CIP, RAC3, and TRAM-1) [1]. Each member of the NCOA family can enhance transcriptional activity of NRs by acting as a bridging molecule, assisting protein-protein interactions between NRs and multiple other co-regulatory factors thereby facilitating the assembly of the transcriptome complex at the target gene promoter [2, 3]. Since coactivators modulate the transcriptional activity of transcription factors, they exert broad genome-wide effects on gene expression networks and contribute significantly to a panorama of physiological and pathological processes [4]. Because of their strong connection with NRs, NCOA proteins have been recognized as key oncoproteins in hormone-dependent cancers [5]. Increased expression of NCOA3 and NCOA1 has been reported in prostate cancers, and their high expression level is associated with tumour grade and disease recurrence [6]. Transcript levels of all three NCOAs were significantly increased in endometrial carcinoma and NCOA3 expression was correlated with clinical stage and poor prognosis [7]. NCOA3 gene is amplified in ovarian cancer and elevated NCOA3 expression has been reported in 64% of high-grade ovarian cancers, and its levels are associated with tumour progression [8]. NCOA3 was found to be overexpressed in >60% of primary breast tumours; however its gene is amplified in only 5%–10% of breast cancers [9, 10]. Transgenic mice overexpressing NCOA3 shows increased mammary epithelial cell proliferation, development of mammary hyperplasia and tumorigenesis [5, 11]. The ablation of NCOA3 in mouse mammary tumour virus (MMTV)/v-Ha-ras mice suppresses mammary gland ductal hyperplasia and mammary gland tumorigenesis [12]. NCOA3 not only functions to promote breast cancer development, it also participates in resistance to anti-hormonal therapy [13]. Increased expression of NCOA3 is strongly correlated with shorter disease-free and overall survival in breast cancer [14].

Accumulation of unfolded/misfolded proteins in the endoplasmic reticulum (ER) activate a set of signalling pathways termed as the Unfolded Protein Response (UPR). This concerted and complex cellular response is mediated by three molecular sensors, PKR-like ER kinase (PERK), activated transcription factor 6 (ATF6), and Inositol-requiring enzyme 1 (IRE1) present in the membrane of ER [15]. The most salient feature of UPR is to increase the transactivation function of a range of transcription factors [16]. Once activated, these transcription factors coordinate transcriptional induction of genes encoding for endoplasmic reticulum-resident chaperones, endoplasmic reticulum-associated degradation (ERAD) machinery, and several others, including many that have no obvious direct relationship to secretory pathway function [17]. We have recently shown that expression of NCOA3 is regulated by XBP1 during the conditions of UPR, as well as estrogen stimulation in human breast cancer cells [18]. Our results described an important non-nuclear receptor (NR) function of NCOA3 where IRE1-XBP1 dependent upregulation of NCOA3 regulates optimal activation of PERK-ATF4 axis during UPR. How NCOA3 regulates PERK-ATF4 signalling during UPR is not clearly understood?

NCOAs are an attractive therapeutic target for treatment of a wide range of hormone-dependent and independent cancers [19]. Unlike protein kinases which are relatively easy to target, coactivators are more difficult to target with small molecules because of their large size, flexible structure and their reliance on protein-protein interactions. Approaches to block coactivator molecules include targeting the enzymes that modulate the stability of NCOAs by posttranslational modifications [20]. However, recent high-throughput screening efforts have identified small-molecule inhibitors that can interact with these coactivators and promote their degradation. Some of the reported inhibitors of NCOAs (Gossypol, Verrucarin A, Bufalin and SI-2) promote the degradation of NCOAs and attenuate cancer cell growth *in vitro* and *in vivo* [19–21]. Surprisingly high-throughput screening also identified MCB-613, a small molecule stimulator of NCOA3 [22]. Counter-intuitively, over-stimulation of NCOAs by MCB-613 leads to death of cancer cells by the generation of reactive oxygen species, ER stress and unfolded protein response [22]. Further MCB-613 kills a broad range of human cancer cells and inhibits tumour growth in a mouse model of breast cancer [22]. However, the molecular determinants of MCB-613-induced cell death are not well defined.

In this study we show that PERK signalling pathway plays a crucial role in MCB-613 mediated cell death. We show that NCOA3 is required for optimal signalling via the PERK pathway during UPR. We show that NCOA3 regulates the expression of PERK and expression levels of NCOA3 mRNA correlates with the transcript levels of PERK in breast cancer cohort of TCGA. Cells lacking NCOA3 show increased sensitivity to ER stress mediated cell death. Hyperstimulation of NCOA3 by MCB-613 leads to enhanced PERK signalling, which contributes to MCB-613 induced cell death. Taken together our results point to NCOA3 as an important determinant in regulating cell fate during ER stress, with too little and too much NCOA3 both producing deleterious effects.

## RESULTS

### NCOA3 modulates integrated stress response during UPR

**Figure 1:**
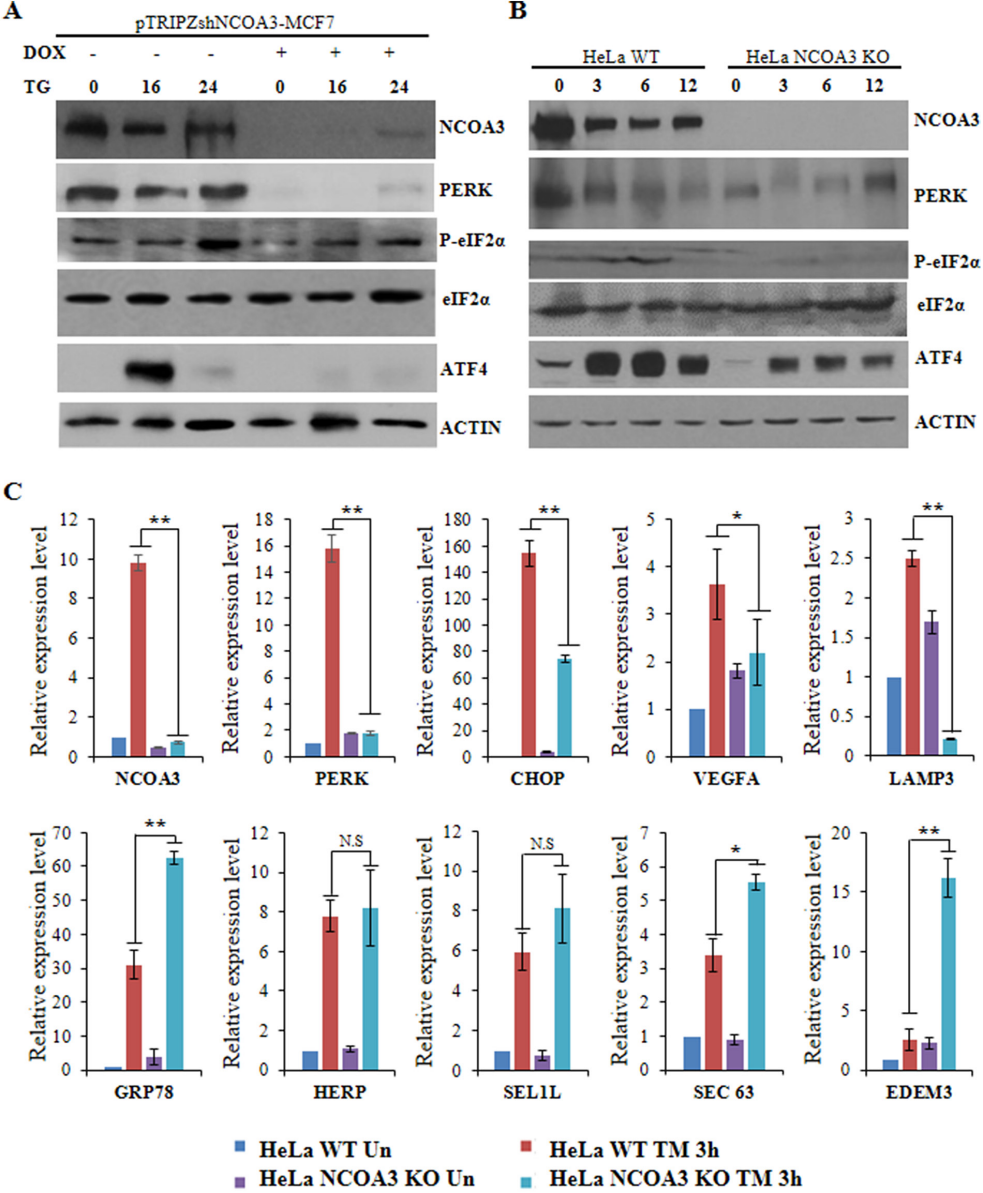
NCOA3 is required for optimal activation of PERK signalling during UPR **(A)** pTRIPZshNCOA3-MCF7 cells were either untreated or treated with TG (1.0 μM) for indicated time points in absence and presence of doxycycline (500 ng/ml). Western blotting of total protein was performed using antibodies against NCOA3, PERK, p-eIF2α, ATF4 and β-actin. **(B)** HeLa WT and HeLa NCOA3 KO cells were either untreated or treated with TM (1.0 μg/ml) for indicated time points. Western blotting of total protein was performed using antibodies against PERK, eIF2α, p-eIF2α, ATF4, NCOA3 and β-actin. **(C)** HeLa WT and HeLa NCOA3 KO cells were either untreated or treated with TM (1.0 μg/ml) for 3 h. Cells were harvested for total RNA isolation at the indicated time points followed by the quantification of the expression of the indicated genes by real-time RT-PCR, normalizing against RPLP0. Results are represented as mean ± S.D. from three independent experiments performed in triplicate. N.S not significant, ^*^P < 0.05, two-tailed unpaired t-test; ^**^ P < 0.01, two-tailed unpaired t-test.

We have previously shown that NCOA3 plays an important role in optimal induction of UPR target genes downstream of the PERK–eIF2α–ATF4 pathway [18]. To understand the mechanisms by which NCOA3 modulates the activation of PERK–eIF2α–ATF4 pathway we evaluated the expression of proximal mediators of PERK signalling during conditions of UPR in presence and absence of NCOA3. For this purpose we used the clones of MCF7 cells expressing tetracycline-inducible NCOA3 shRNA (pTRIPZ-shNCOA3-MCF7) where addition of doxycycline reduces the expression of NCOA3 protein [18]. We evaluated the expression of PERK, phospho-eIF2α and ATF4 proteins in pTRIPZ-shNCOA3-MCF7 cells that were either untreated or treated with (TG) thapsigargin in absence and presence of doxycycline. As expected we observed significant knockdown of NCOA3 protein after the addition of (500 ng/ml) doxycycline to pTRIPZ-shNCOA3-MCF7 clone (Figure 1A). We observed that constitutive expression of PERK protein and (TG) thapsigargin-mediated induction of phospho-eIF2α and ATF4 was compromised in absence of NCOA3 (Figure 1A). Next, we used the NCOA3 (KO) knockout and (WT) wild-type HeLa cells to evaluate the effect of NCOA3 on PERK signalling. HeLa cells that lack a functional copy of the NCOA3 gene were generated using a zinc finger nuclease that targets exon 11 of the NCOA3 gene [21]. We found that basal expression of PERK protein and (TM) tunicamycin-mediated induction of phospho-eIF2α and ATF4 was compromised in NCOA3 KO HeLa cells as compared to WT HeLa cells (Figure 1B). In agreement with its effect PERK-eIF2α-ATF4 axis, TM-mediated induction of UPR target genes down stream of PERK axis (CHOP, VEGFA and LAMP3) were attenuated in NCOA3 KO as compared to WT HeLa cells (Figure 1C). There was no difference in the induction of HERP and SEL1L while the induction of GRP78, SEC63 and EDEM3 was augmented in NCOA3 KO as compared to WT HeLa cells (Figure 1C). These results suggest that NCOA3 is required for the optimal activation of PERK-eIF2α-ATF4 pathway during conditions of ER stress.

### NCOA3 regulates the expression of PERK

**Figure 2:**
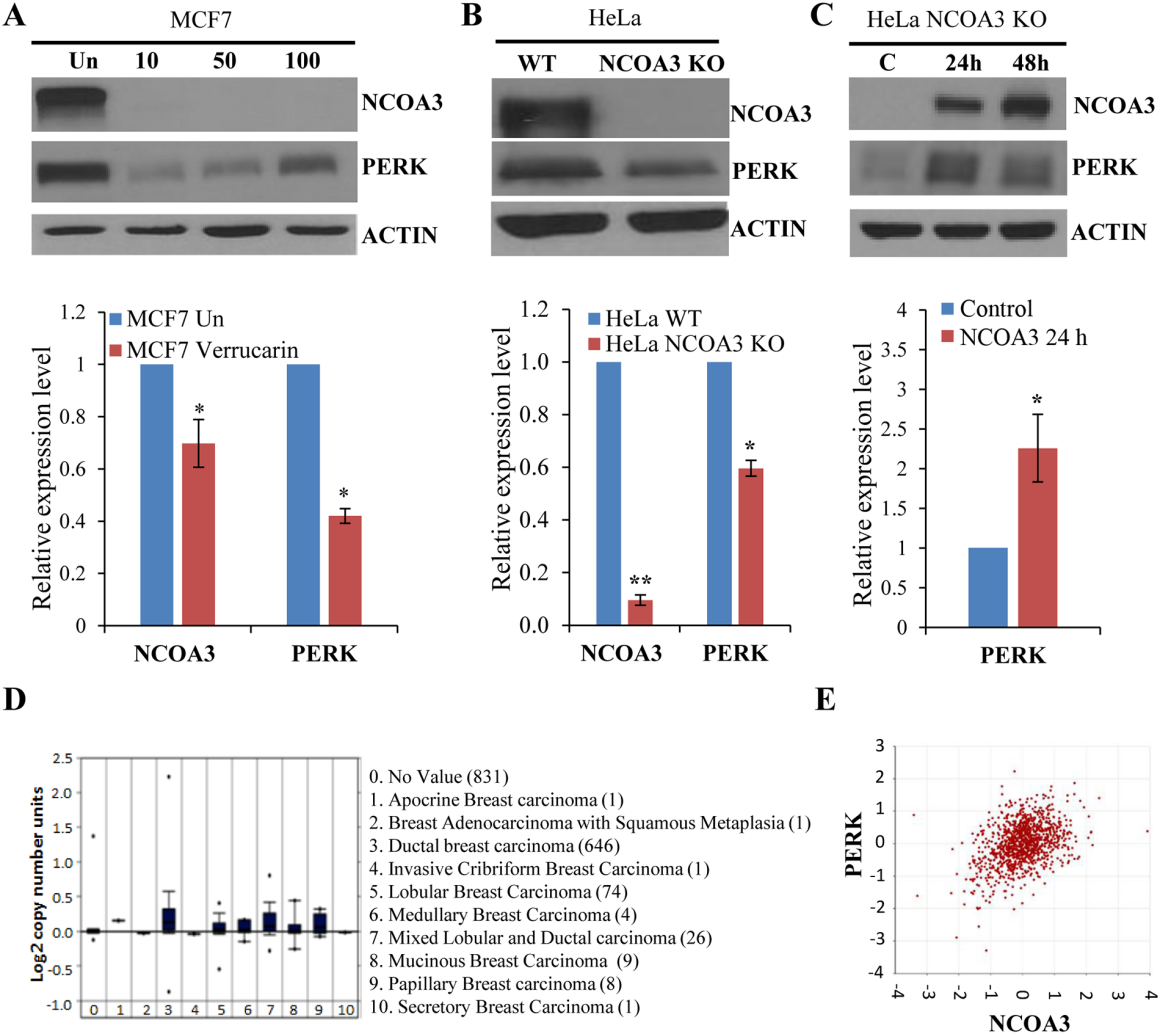
NCOA3 regulates the expression of PERK **(A)** MCF7 cells were treated with Verrucarin A at different concentrations (10 nM, 50 nM, and 100 nM) for 24 h. Upper panel, western blotting of total protein was performed using antibodies against NCOA3, PERK and β-actin. Lower panel, the expression level of NCOA3 and PERK was quantified by real-time RT-PCR normalizing against RPLP0. Results are represented as mean ± S.D. from three independent experiments performed in triplicate. **(B)** Upper panel, western blotting of total protein isolated from HeLa WT and HeLa NCOA3 KO cells was performed using antibodies against NCOA3, PERK and β-actin. Lower panel, expression level of NCOA3 and PERK was quantified using total RNA isolated from HeLa WT and HeLa NCOA3 KO cells by real-time RT-PCR normalizing against RPLP0. Results are represented as mean ± S.D. from three independent experiments performed in triplicate. **(C)** HeLa NCOA3 KO cells were transiently transfected with NCOA3 overexpressing plasmid for indicated time points. Upper panel, western blotting of total protein was performed using antibodies against NCOA3, PERK and β-actin. Lower panel, expression level of NCOA3 and PERK was measured by real-time RT-PCR normalizing against RPLP0. Results are represented as mean ± S.D. from three independent experiments performed in triplicate. **(D)** Box plots derived from copy number alteration in Oncomine comparing the alteration of NCOA3 gene in the indicated categories of breast adenocarcinoma are shown. **(E)** The dot plot of log2 transformed values for co-expression of NCOA3 and PERK as determined by R2 is shown. ^*^P < 0.05, two-tailed unpaired t-test; ^**^ P < 0.01, two-tailed unpaired t-test.

Next, we used a recently identified small molecule inhibitor of NCOA3, Verrucarin A, which selectively promotes the degradation of the NCOA3 protein [23]. We found that treatment of MCF7 WT cells with Verrucarin A led to decrease in the protein and mRNA level of NCOA3 and PERK (Figure 2A). In agreement with our observations with Verrucarin A, NCOA3 KO HeLa cells showed reduced protein and mRNA levels of NCOA3 and PERK (Figure 2B). Further, we observed that ectopic expression of wild-type NCOA3 in NCOA3 KO HeLa cells rescued the reduced expression of PERK mRNA and protein (Figure 2C). Analysis of copy number alteration (CNA) of NCOA3 in the BRCA-The Cancer Genome Atlas (TCGA) data sets, which is one of the largest data sets containing matched DNA copy number alteration of primary breast cancers revealed amplification of NCOA3 gene (Figure 2D). In agreement with regulation of PERK expression by NCOA3 we found that expression of PERK mRNA levels showed moderate positive relationship with the transcript levels of NCOA3 (Significance of correlation: R-value=0.391, p-value=2.5e-41, T-value=14.048, degrees of freedom=1095) in BRCA-TCGA datasets of breast cancer patients (Figure 2E). We observed that half-life of PERK protein and mRNA was not significantly different in NCOA3 KO and WT HeLa cells (Supplementary Figure 1). These results suggest that NCOA3 regulates PERK expression most likely by acting as a coactivator required for basal transcription of PERK.

### NCOA3-deficient HeLa cells are hypersensitive to ER stress mediated cell death

**Figure 3:**
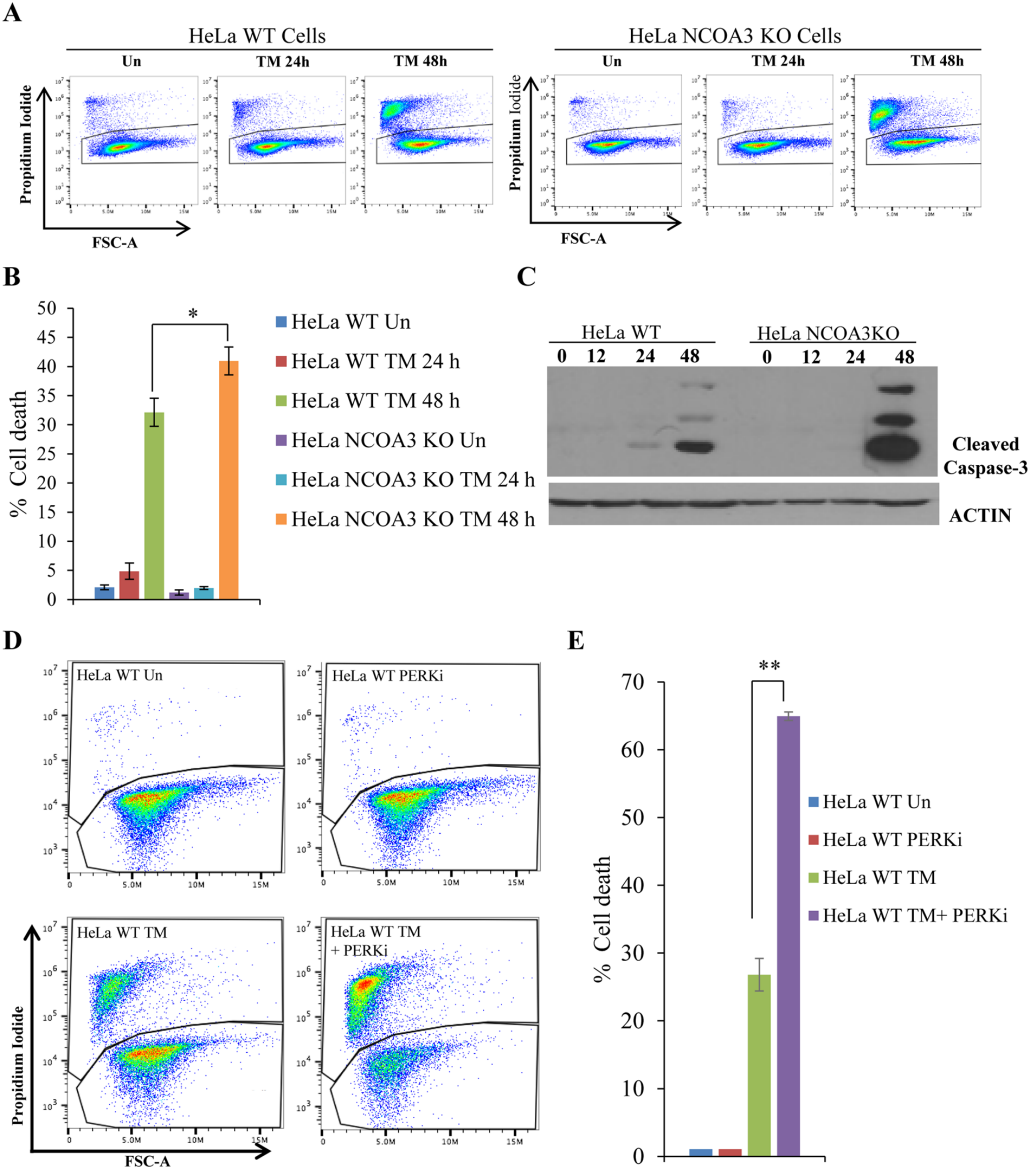
NCOA3-deficient HeLa cells are hypersensitive to ER stress mediated cell death **(A)** HeLa WT and HeLa NCOA3 KO cells were either untreated or treated with TM (1 μg/ml) for 24 h and 48 h. Representative dot plot of propidium iodide (PI) staining of Hela WT and Hela NCOA3 KO cells are shown. **(B)** HeLa WT and HeLa NCOA3 KO cells staining positive for PI are shown as dead cells (n=3). **(C)** HeLa WT and HeLa NCOA3 KO cells were treated as in A, and western blotting of total protein was performed using antibodies against cleaved caspase-3 and β-actin. **(D)** Representative dot plot of PI staining of HeLa WT cells treated with TM (1 μg/ml) for 48 h in absence or presence of 100 nM (PERKi) GSK PERK inhibitor (n=3). **(E)** Cells were treated as in D, PI positive are shown as dead cells (n=3). ^*^P < 0.05; ^**^ P < 0.01 (two-tailed unpaired t-test).

During UPR, activated PERK phosphorylates eukaryotic translation initiation factor2α (eIF2α), which reduces the global protein synthesis [24]. The PERK-dependent reduction of protein translation limits nascent protein transport to ER lumen thereby reducing the client protein load on the ER [25]. Mouse embryonic fibroblasts (MEFs) from PERK knockout mice and mice expressing homozygous mutation at the eIF2α phosphorylation site (Ser51Ala) are hypersensitive to ER stress-induced apoptosis [26]. We observed that NCOA3 KO HeLa cells showed increased sensitivity to ER stress induced cell death as compared to WT HeLa cells (Figure 3A, 3B). The expression of cleaved caspase-3, a marker of apoptotic cell death, was induced robustly in HeLa NCOA3 KO cells upon TM treatment as compared to HeLa WT cells (Figure 3C). Further we found that GSK PERK inhibitor sensitized the WT HeLa cells to ER stress-induced cell death (Figure 3D, 3E). These results suggest that attenuated PERK signalling in NCOA3 KO HeLa cells may be responsible for their hypersensitivity to ER stress-mediated cell death.

### MCB-613 induced cell death is dependent on NCOA3

**Figure 4:**
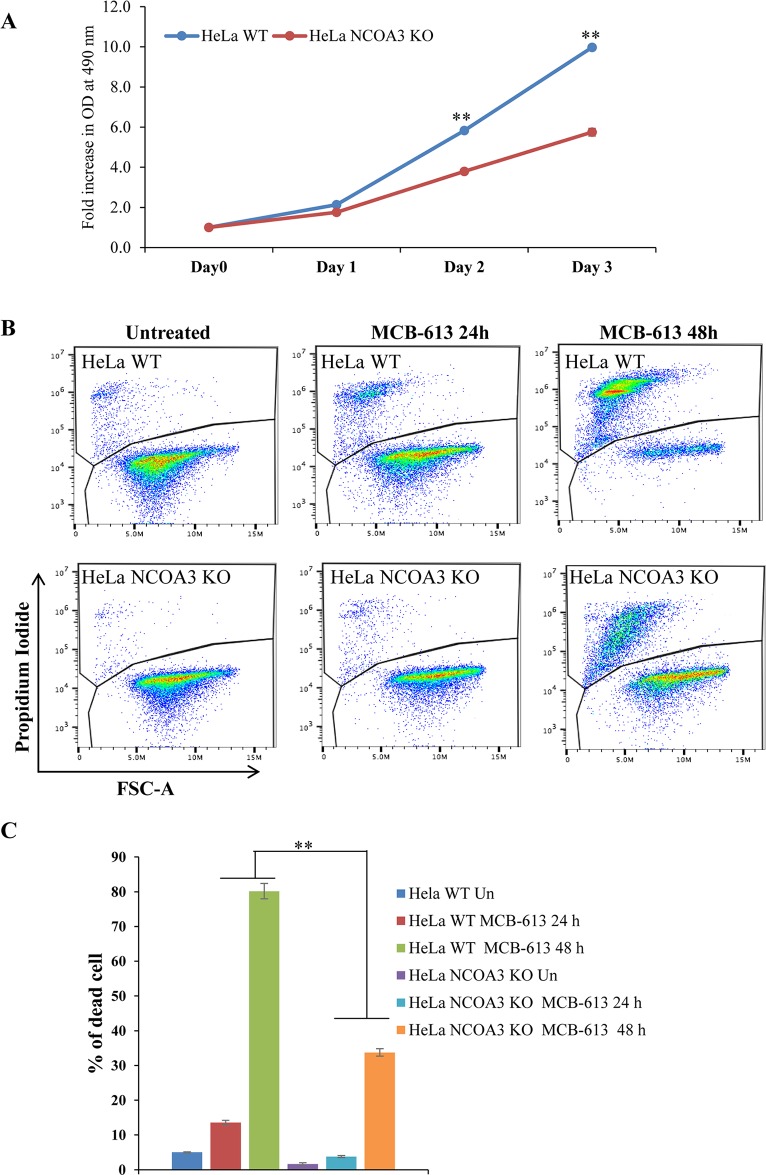
NCOA3-deficient HeLa cells are resistant to MCB-613 mediated cell death **(A)** HeLa WT and HeLa NCOA3 KO cells were plated in 96-well plate (2,000 cells/well). Line graphs show the fold change absorbance at the indicated time points after the treatment. Error bars represent mean ± S.D. from three independent experiments performed in triplicate. **(B)** HeLa WT and HeLa NCOA3 KO cells were treated with MCB-613 (10 μM) for 24 h and 48 h. Representative dot plot of PI staining of HeLa WT and HeLa NCOA3 KO cells are shown. **(C)** HeLa WT and HeLa NCOA3 KO cells staining positive for PI are shown as dead cells (n=3). ^*^P < 0.05; ^**^ P < 0.01 (two-tailed unpaired t-test).

MCB-613 is a Pan-NCOA stimulator [22]. We used the NCOA3 KO and WT HeLa cells to evaluate the role of NCOA3 in MCB-613 induced cell death. We were able to confirm the previously published observations that loss of NCOA3 reduced the proliferation of HeLa cells (Figure 4A) and NCOA3 KO HeLa cells showed reduced sensitivity to MCB-613 as compared to WT HeLa cells (Figure 4B, 4C). These results suggest that cell death induced by MCB-613 is at least partially dependent on NCOA3 (NCOA1 and NCOA2 are still expressed in NCOA3 KO HeLa cells).

### MCB-613 leads to enhanced activation of PERK signalling and induces PERK dependent cell death

**Figure 5:**
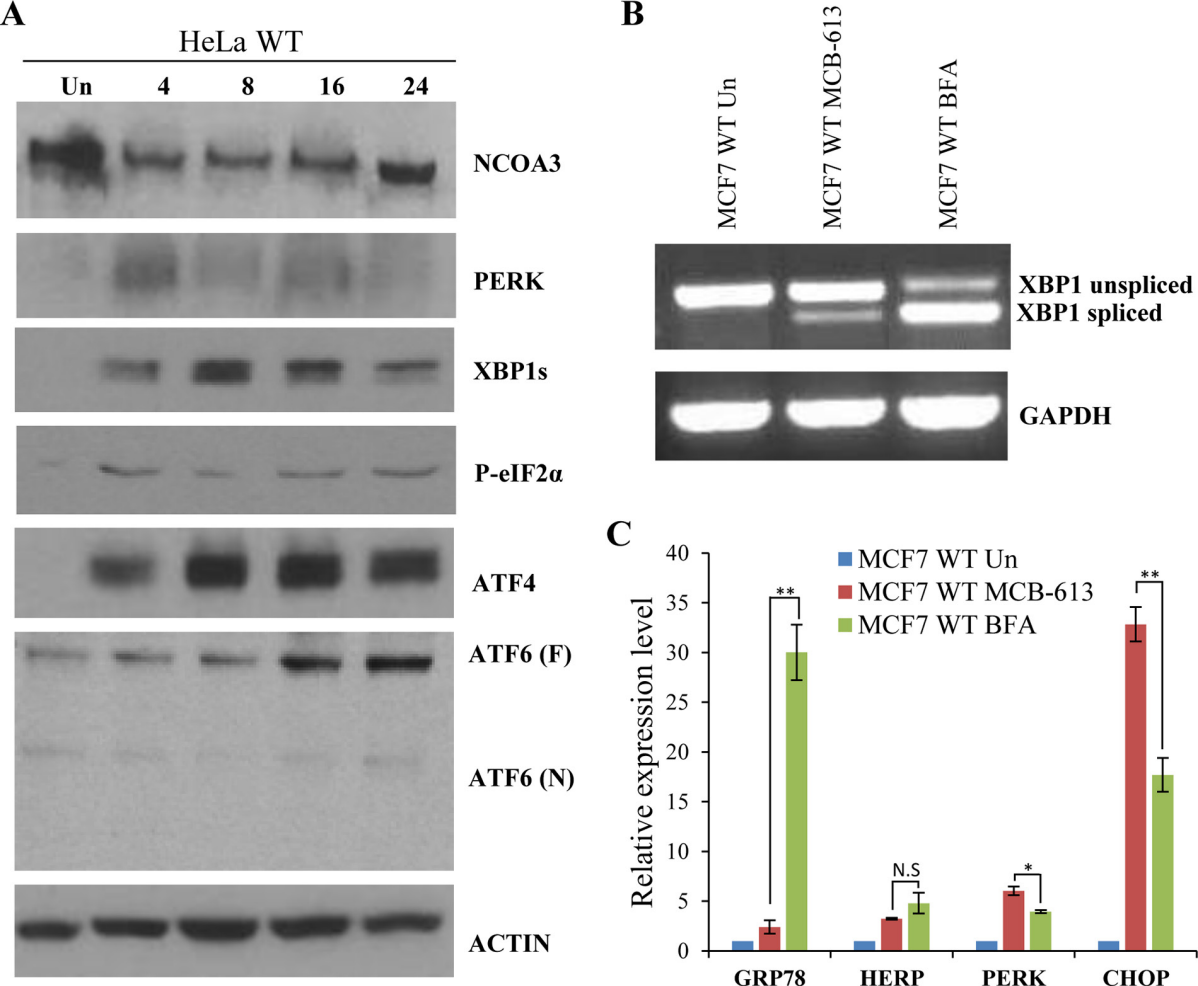
MCB-613 leads to enhanced activation of PERK signalling **(A)** HeLa WT cells were either untreated or treated with MCB-613 (10 μM) for 4 h, 8 h, 16 h and 24 h. Western blotting of total protein was performed using antibodies against UPR target proteins (PERK, XBP1s, p-eIF2α, ATF4, ATF6), NCOA3 and β-actin. **(B-C)** MCF7 cells were treated with MCB-613 (5 μM) or BFA (1 μg/ml) for 24 h. The expression level of XBP1 was measured by conventional PCR and the expression of UPR target genes (GRP78, HERP, PERK, and CHOP) was quantified by real-time RT-PCR, normalizing against RPLP0. Results are represented as mean ± S.D. from three independent experiments performed in triplicate. N.S not significant, ^*^P < 0.05, two-tailed unpaired t-test; ^**^ P < 0.01, two-tailed unpaired t-test.

MCB-613 treatment leads to ER stress and UPR downstream of hyperactivation of NCOAs, resulting in paraptotic cell death [22]. NCOA3 activation is coupled to its turnover and hyperactivation of NCOA3 by MCB-613 is accompanied by reduction in total NCOA3 protein [22]. In agreement with previous results, we found that treatment with MCB-613 resulted in decrease in NCOA3 protein levels (Figure 5A). We found that MCB-613 treatment leads to induction of multiple UPR markers such as PERK, XBP1s, phospho-eIF2α, ATF4 and ATF6 (Figure 5A). Next we compared the induction of UPR target genes upon treatment with MCB-613 and Brefeldin A (BFA). BFA reversibly inhibits anterograde transport from the endoplasmic reticulum to the Golgi apparatus leading to UPR [27]. We found that both MCB-613 and BFA induced the expression of spliced XBP1, GRP78, HERP, PERK and CHOP (Figure 5B, 5C). We found that BFA was more potent and robust inducer of spliced XBP1 and GRP78 as compared to MCB-613 whereas MCB-613 treatment showed significantly higher induction of PERK and CHOP (Figure 5B, 5C). These results indicate that MCB-613 treatment induces UPR with augmented activation of PERK signalling.

**Figure 6:**
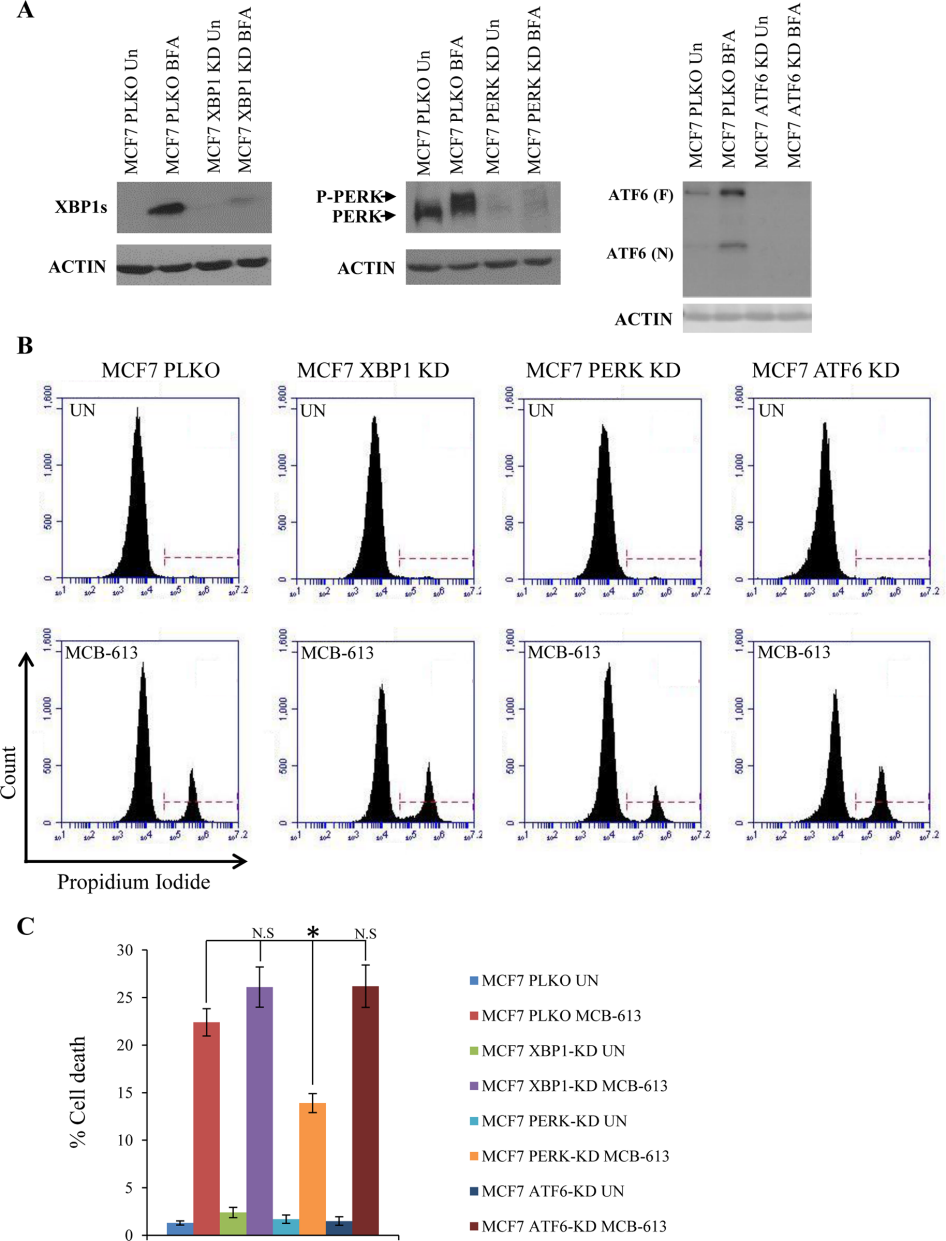
MCB-613 induces PERK dependent cell death **(A)** MCF7-control (PLKO), MCF7-XBP1 knockdown (XBP1-KD) and MCF7 PERK knockdown (PERK-KD) and MCF7-ATF6 knockdown (ATF6-KD) cells were treated with (1.0 μg/ml) Brefeldin A (BFA) for 18 h. Western blotting of total protein was performed using the indicated antibodies. **(B)** MCF7-control (PKLO), MCF7-XBP1 knockdown (XBP1-KD), MCF7 PERK knockdown (PERK-KD) and MCF7-ATF6 knockdown (ATF6-KD) cells were either untreated or treated with (10 μM) MCB-613 for 24 hr. Representative dot plot of PI staining of cells are shown. **(C)** MCF7 PKLO, MCF7 XBP1-KD, MCF7 PERK-KD and MCF7 ATF6-KD cells staining positive for PI are shown as dead cells (n=3). N.S not significant, ^*^P < 0.05, two-tailed unpaired t-test.

Genetic and pharmacological experiments have demonstrated that PERK signalling can confer both protective and proapoptotic outcomes in the face of ER stress [15]. In particular, during chronic ER stress persistent PERK activity contributes to ER stress-induced cell death [28]. Next, we evaluated whether enhanced PERK signalling plays a role in MCB-613 mediated cytotoxicity. To determine the role of UPR sensors (XBP1, PERK and ATF6) in MCB-613 mediated cell death, we generated the control (MCF7-PLKO), XBP1 knockdown (MCF7 XBP1-KD), PERK knockdown (MCF7 PERK-KD) and ATF6 knockdown (MCF7 ATF6-KD) subclones of MCF7 cells. We observed the reduction in the (basal and TG-induced) expression of cognate target gene due to the expression of the corresponding shRNA (Figure 6A). We found that MCB-613 induced cell death was specifically attenuated in MCF7 PERK-KD cells (Figure 6B, 6C) as compared to MCF-PLKO cells, while knockdown of XBP1 and ATF6 had no effect. Collectively, these results suggest MCB-613 leads to enhanced PERK signalling which contributes at least in part to MCB-613 mediated cell death.

## DISCUSSION

Nuclear receptor coactivators have emerged as “master regulators” of human homeostasis, and their dysregulation has been implicated in several diseases [4]. The involvement of NCOAs family members in cancer, reproduction and energy metabolism underscores their importance in pathophysiology of human diseases [2]. Our results reveal a novel non-NR role for NCOA3 in UPR signalling, where NCOA3 plays an important role in optimal activation of PERK-eIF2α-ATF4 pathway (Figure 1). PERK-ATF4 arm directly upregulates vascular endothelial growth factor A (VEGFA) and Lysosomal-Associated Membrane Protein 3 (LAMP3) thereby regulating tumour vascularity and invasion [29, 30]. Indeed, loss of NCOA3 attenuated the UPR-mediated increase in the expression of VEGFA and LAMP3 (Figure 1). The UPR signalling pathways are important for normal cellular homeostasis and play key roles in the pathogenesis of many diseases [15]. Accumulating evidence incriminate UPR-induced cellular dysfunction and cell death as major contributors to pathology of diseases, making modulators of UPR pathways an attractive targets for therapeutic interventions [16, 31, 32]. Our results provide a rationale for evaluating the recently described NCOAs targeting compounds to modulate UPR pathways.

Although NCOAs were identified as coactivators for NR-dependent transcription, they have been shown to interact with many different transcription factors and potentiate their transcriptional activity [2]. NCOAs provide important scaffold for the assembly of transcription machinery at the gene promoter thereby modulating the transcriptional activity of the promoter. We show that NCOA3 regulates the expression of PERK mRNA and protein without altering their turnover (Supplementary Figure 1). Our results suggest that NCOA3 regulates PERK expression by acting as a coactivator for transcription factor(s) required for basal transcription of PERK. In addition to NRs, NCOA3 act as coactivator for several other transcription factors [AP-1, PEA3, NF-κB, E2F1, SMADs, HIF1, TP53, STATs, ETS and HNF4] to control diverse gene regulatory networks [1, 2]. Indeed, bioinformatics analysis has shown the presence of binding sites for AP1 and NF-kB sites (TFs known to interact with NCOA3) in the promoter region of PERK. In line with regulation of PERK expression by NCOA3 we found enhanced activation of PERK-ATF4 signalling pathway upon stimulation of NCOA3 by MCB-613 treatment (Figure 5). UPR stress sensors have been shown to be activated in a selective manner, such as specific activation of IRE1 by Toll-like receptors in macrophages [33], specific activation of PERK during epithelial to mesenchymal transition [34] and specific activation of ATF6 by ER membrane protein overload [35].

**Figure 7:**
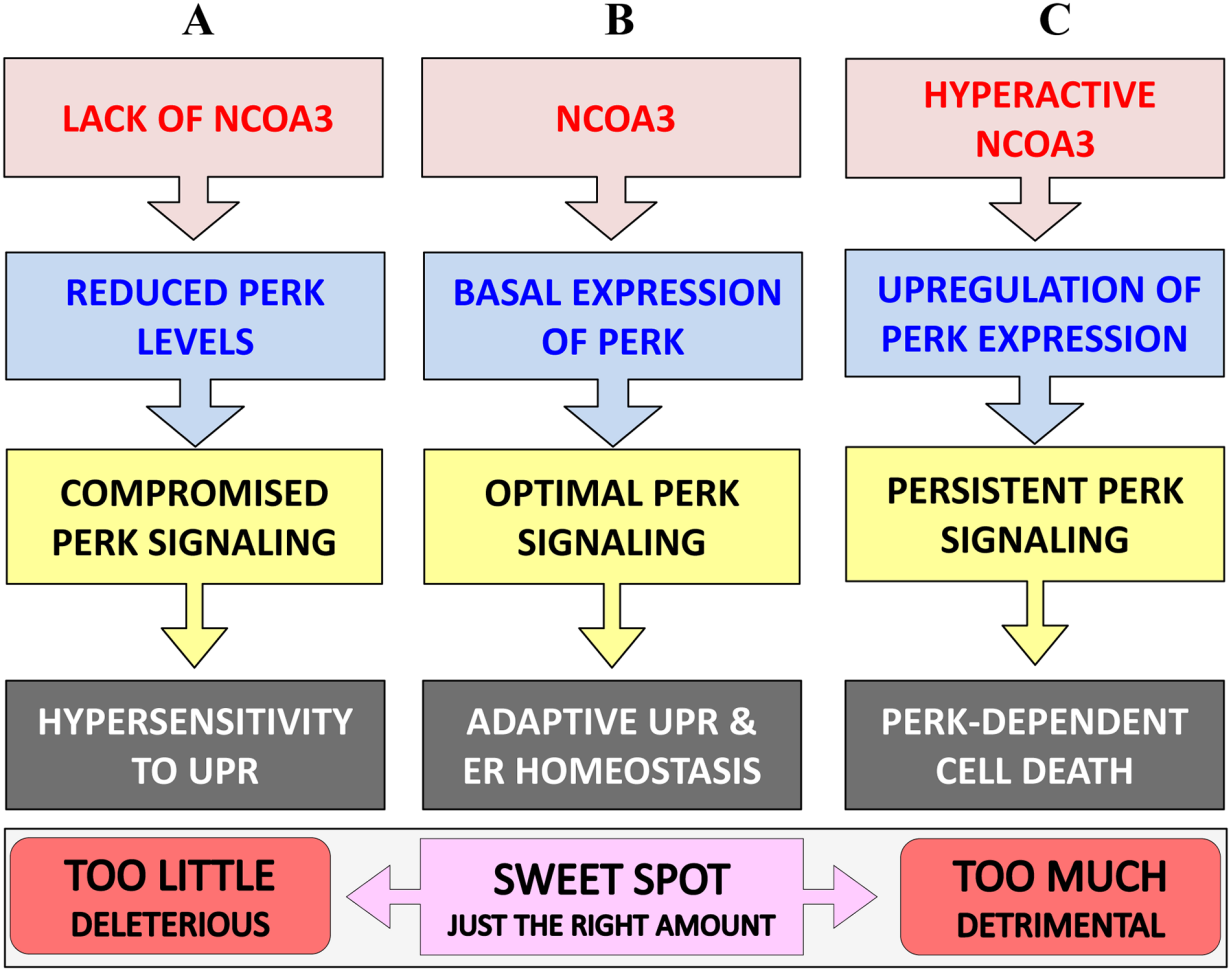
Graphical summary **(A)** Loss of NCOA3 leads to reduced PERK expression and attenuated PERK signalling during UPR which in turn renders cells hypersensitive to ER stress-mediated cell death. **(B)** During the normal physiological conditions NCOA3 maintains the basal expression of ER stress sensor, PERK. The basal expression of PERK is essential for optimal PERK signalling and restoring ER homeostasis during the conditions of UPR. **(C)** Hyper activation of NCOA3 leads to increased expression of PERK and PERK-dependent cell death. Taken together our results point to NCOA3 as an important determinant in regulating cell fate during ER stress, with too little and too much NCOA3 both producing deleterious effects.

PERK signalling mediates both adaptive, as well as apoptotic, responses depending on the intensity and duration of the stress, it may promote, as well as suppress, cell death depending on the context [26, 36]. In our model, NCOA3 regulated expression of PERK is required for optimal PERK signalling during UPR, with too little and too much NCOA3 both producing deleterious effects (Figure 7). We found that loss of NCOA3 or co-treatment with PERK inhibitor sensitized HeLa cells to ER stress-induced apoptosis which is most likely due to compromised PERK signalling (Figure 3). Indeed PERK^−/−^ and ATF4^−/−^ MEFs and eIF2α (Ser51Ala) knock-in MEFs are hypersensitive to ER-stress-induced apoptosis [37, 38]. Further liver-specific loss of PERK has been shown to increase ER-stress induced apoptosis in an animal model [39]. Our observation showing that persistent PERK activity upon MCB-613 treatment impairs cell viability is consistent with previous reports showing that selective activation of PERK leads to cell death [40]. Persistent PERK signalling could impair cell viability via induction of CHOP, a transcription factor that has been shown to contribute to ER stress-induced apoptosis *in vitro* and *in vivo* [41, 42]. Taken together our findings suggest that the duration and/or intensity of PERK signalling downstream of NCOA3 is a key determinant of regulation of cell fate by NCOA3 during UPR (Figure 7).

Several studies have shown that NCOAs play an important role in biology of hormone-dependent and independent cancers. Both *in vitro* and *in vivo* studies have demonstrated that NCOA3 is involved in many cancer processes through several mechanisms [2, 43]. Such results have stimulated attempts to generate small molecules that modulate activity of NCOAs [19, 20]. Pharmacological hyperactivation of NCOA3 by MCB-613 has been shown to attenuate cancer cell growth *in vitro* and *in vivo* [22]. Our results provide novel insights into the mechanism of MCB-613 induced cell death whereby MCB-613 leads to enhanced PERK signalling which contributes to MCB-613 mediated cell death (Figure 7). More importantly we report that reduction of PERK expression provides resistance to MCB-613 mediated cell death (Figure 6). PERK has been shown to be a haploinsufficient tumour suppressor in melanomas where mono-allelic deletion of PERK is required for tumour progression [44]. Furthermore hypomorphic mutants of PERK (A422V, T428A, H436Y, Y474C, P483Q, P993R, 911fs) have been reported to act as dominant inhibitor of wild-type PERK and contribute to the progression of melanoma [44]. Genomic Data Commons Data Portal at National Cancer Institute lists 254 mutations spread throughout PERK coding exons and distributed across 22 different types of cancers. Interestingly the dominant negative mutations of PERK identified in human melanomas [44] are also present in several other human cancers. As the efforts to develop small molecule stimulators of nuclear receptor coactivators for cancer treatment progress, an open question emanates from our work about the role of hypomorphic PERK mutations in providing resistance to such therapy.

## MATERIALS AND METHODS

### Cell culture and treatments

MCF7 cells were purchased from ECACC. Wild type and NCOA3 knockout HeLa cells were a kind gift from Bert O’Malley, Baylor College of Medicine, USA. HEK 293T cells were from Indiana University National Gene Vector Biorepository. Cells maintained in Dulbecco’s modified medium (DMEM) supplemented with 10% FBS, 100 U/ml penicillin and 100 mg/ml streptomycin at 37°C with 5% CO2. To induce ER stress, cells were treated with tunicamycin (TM), thapsigargin (TG) or Brefeldin A (BFA) at the indicated concentrations for the indicated time. Thapsigargin (Cat # 1138), tunicamycin (Cat # 3516), BFA (Cat # 1231) were sourced from Tocris Bioscience. MCB-613 (SML1567) was obtained from Sigma-Aldrich, Ireland. To inhibit PERK activity cells were treated with GSK2606414 (Cat # 516535 Millipore Ireland B.V).

### Plasmid constructs

The PERK shRNA expressing lentiviral plasmid was a kind gift from Dr. Piyush Gupta, Massachusetts Institute of Technology at Boston, USA. The ATF6 shRNA expressing lentiviral plasmid (TRCN0000017853, TRCN0000017855 and TRCN0000017857) were purchased from Dharmacon GE Healthcare Life Sciences. The expression vector for FLAG-tagged NCOA3 was a kind gift from Dr. Hongwu Chen, University of California at Davis, USA.

### Generation of stable cell lines

The MCF7 XBP1-KD and pTRIPZshNCOA3-MCF7 cells have been described previously [18]. Lentivirus expressing PERK shRNA and ATF6 shRNA were generated by transfecting lentiviral plasmids along with packaging plasmids in 293T cells using jetPEI transfection reagent (Polyplus transfection, VWR International Ltd, Dublin, Ireland) according to manufacturer’s instructions. MCF7 cells were then transduced with the shRNA lentivirus and selection for shRNA-positive cells was performed with 2 μg/ml puromycin for 7 days.

### RNA extraction, RT-PCR and real time RT-PCR

Total RNA was isolated using Trizol (Life Technologies) according to the manufacturer’s instructions. Reverse transcription (RT) was carried out with 2 μg RNA and random primers (Promega) using ImProm-II™ Reverse Transcription System (Promega). Real-time PCR method to determine the induction of UPR target genes has been described previously [45].

### MTS cell proliferation assay

CellTiter 96® Aqueous One Solution Cell Proliferation Assay (Promega; Cat. No: G1112) reagent was used in this assay procedure. Cells were (HeLa WT and HeLa NCOA3 KO) seeded in 96-well flat bottom clear plate (2,000 cells/well). For each subclones, ten replicates were set for one sample. Plates were incubated in CO_2_ humidified incubator at 37°C for overnight to attach the cells. On the day of MTS test, 100 μl PMS (0.9 mg/ml) (Sigma-Aldrich; Cat. No: 78830-1G) was added to 1 ml of MTS reagent (2 mg/ml) immediately before addition to the plate. At set time points (Day 0, 1, 2, 3) 20 μl of the premixed MTS+PMS reagent was added and measurements were made in accordance with the manufacturer’s instructions (Promega Corp., Madison, WI, USA).

### Western blotting

Western blotting procedures has been described previously [46]. The primary antibodies used were NCOA3 (Cell signalling, Cat# 2126), ATF6 (Abcam, Cat# ab122897), spliced XBP1 (Biolegend, Cat# 619502), PERK (Cell signalling, Cat# C33E10), phospho-eIF2α (Cell signalling, Cat# 9721), total eIF2α (Cell signalling, Cat# 9722), ATF4 (Santa Cruz Biotechnology, Cat# sc-200), cleaved caspase-3 (Cell Signalling, Cat# 9661) and or β-Actin (Sigma, Cat# A-5060) overnight at 4°C. The membrane was washed 3 times with PBS-0.05% Tween or TBS-0.1% Tween (total-eIF2α and phospho-eIF2α) where appropriate and further incubated in appropriate horseradish peroxidase-conjugated secondary antibody (Pierce) for 2h at room temperature. Signals were detected using Western Lightening Plus ECL (Perkin Elmer).

### Propidium iodide staining

Membrane permeability was assessed using propidium iodide staining. Briefly, cells were harvested by trypsinization, membrane integrity was allowed to be restored for 15 min at 37°C. Cells were collected by centrifugation, resuspended in PBS. Cells were stained with 0.4 μg/ml of PI and analysed using BD Accuri C6 flow cytometer (Becton Dickinson). Flow cytometric results were analysed using FlowJo Software (V10).

### Patient samples and data analysis

Patient data were assessed from TCGA corresponding to breast cancer samples with available whole-genome DNA copy number alterations, mRNA expression data, and clinicopathological data. The copy number alteration of NCOA3 was analysed from the BRCA-TCGA data set using Oncomine [47]. For this, we compared clinical specimens of cancer vs. normal patient datasets. In order to reduce our false discovery rate, we selected p<0.01 as a threshold. The co-expression of PERK and NCOA3 in BRCA-TCGA data set was analysed using R2 (R2: Genomics Analysis and Visualization Platform http://r2.amc.nl).

### Statistical analysis

The data is expressed as mean ± SD for three independent experiments. Differences between the treatment groups were assessed using Two-tailed paired student’s t-tests. The values with a p<0.05 were considered statistically significant.

## SUPPLEMENTARY MATERIALS FIGURES AND TABLES



## Abbreviations

NCOAs: Nuclear receptor coactivators
NCOA3: Nuclear receptor coactivator 3
UPR: Unfolded protein response
NR: Nuclear receptor
MMTV: Mouse mammary tumour virus
ER: Endoplasmic Reticulum
PERK: PKR-like ER kinase
ATF6: Activated transcription factor 6
IRE1: Inositol-requiring enzyme 1
ERAD: Endoplasmic reticulum-associated degradation
TG: Thapsigargin
TM: Tunicamycin
BFA: Brefeldin A
TCGA: The Cancer Genome Atlas
CNA: Copy number alteration
MEFs: Mouse embryonic fibroblasts
VEGFA: Vascular endothelial growth factor A
LAMP3: Lysosomal-Associated Membrane Protein 3
shRNA: small hairpin RNA
RT: Reverse transcription
MTS: 3-(4,5-dimethylthiazol-2-yl)-5-(3-carboxymethoxyphenyl)-2-(4-sulfophenyl)-2Htetrazolium, inner salt;
PMS: Phenazine Methosulfate.
