# Supplementary material for: Hyperactivation of nuclear receptor coactivators induces PERK-dependent cell death
Source: Oncotarget. 2018 Feb 8;9(14):11707–21. doi: 10.18632/oncotarget.24451 (PMC5837751; doi:10.18632/oncotarget.24451)
Supplement: Supplementary file 1 [file oncotarget-09-11707-s001.pdf]

Hyperactivation of nuclear receptor coactivators induces PERK-dependent cell death

SUPPLEMENTARY MATERIALS

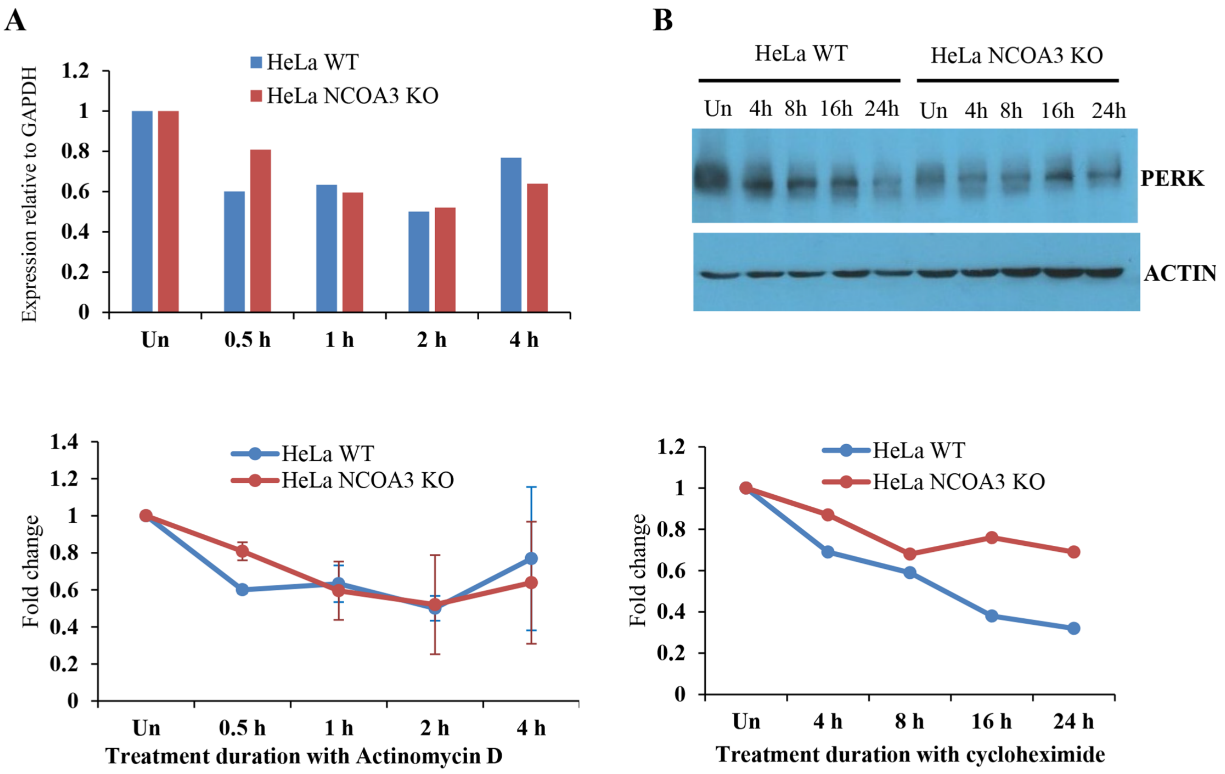

Supplementary Figure 1: Half life of PERK mRNA and protein in HeLa WT and NCOA3 KO cells. (A) HeLa WT and NCOA3 KO cells are treated with Actinomycin D (1 µg/ml) for the indicated time points. The expression level of PERK was quantified by Taqman real time qRT PCR, normalizing against GAPDH. Lower panel, line graphs show the fold change in PERK mRNA at the indicated time points after treatment. **(B)** HeLa WT and NCOA3 KO cells are treated with cycloheximide (100 µg/ml) for the indicated time points. Western blotting of total protein was performed using PERK and β-actin. Autoradiographs were scanned and analysed using Image J software. Lower panel, fold change of the expression total PERK at different time points after treatment with cycloheximide in HeLa WT and Hela NCOA3 KO cells is shown here normalizing against β-actin by considering the untreated value as 1.
